# Multisectoral action towards sustainable development goal 3.d and building health systems resilience during and beyond COVID-19: Findings from an INTOSAI development initiative and World Health Organization collaboration

**DOI:** 10.3389/fpubh.2023.1104669

**Published:** 2023-05-19

**Authors:** Siri Hellevik, Saqif Mustafa, Yu Zhang, Archana Shirsat, Sohel Saikat

**Affiliations:** ^1^INTOSAI Development Initiative, Oslo, Norway; ^2^World Health Organization (Switzerland), Geneva, Switzerland

**Keywords:** health systems resilience, sustainable development goals, supreme audit institutions, public health, health policy, COVID-19, universal health coverage, health security

## Abstract

This article is part of the Research Topic ‘Health Systems Recovery in the Context of COVID-19 and Protracted Conflict’.

As the world faces global health crises such as pandemics, epidemics, climate change and evolving disease burdens and population demographics, building strong and resilient public health systems is of critical importance. The need for an integrated approach to building health system resilience; the widening of inequalities; and fears of vulnerable populations being left behind are critical issues that require Supreme Audit Institutions (SAIs) enquiry as independent public oversight bodies. Each country has a Supreme Audit Institution with a remit to audit public funds as an effective, accountable, and inclusive institution. Government audits are key components of effective public financial management and Good Governance. SAIs contribute to the quality of government engagement and better state-society relations through their work. As SAIs provide independent external oversight and contribute to follow up and review of national targets linked to the Sustainable Development Goals (SDGs) in their respective countries, they can play an important role in national recovery efforts. WHO and INTOSAI Development Initiative (IDI) have been collaborating in facilitating SAIs’ audits of strong and resilient national public health systems linked to the national target of SDG 3.d in 40 countries across Africa, Americas, Asia and Oceania between 2021 and 2022. This paper aims to convey key lessons learned from the joint multisectoral collaboration for facilitating the 3.d audits that can contribute to building health systems resilience in ongoing recovery efforts. The collaboration included facilitation of the audits through professional education and audit support using a health systems resilience framework. The 3.d audits are performance audits and follow IDI’s SDG Audit Model (ISAM). Following the ISAM implies that the SAI should focus on a whole-of-government approach, policy coherence and integration, and assess both government efforts at ‘leaving no one behind’ and multi-stakeholder engagement in implementing the chosen national SDG target linked to 3.d. WHO’s Health Systems Resilience team has supported IDI and SAIs by delivering training sessions and reviewing working papers and draft reports of the SAIs from a health systems resilience perspective. IDI has provided the technical expertise on performance audits through its technical team and through in-kind contributions from mentors from many SAIs in the regions participating in the audit. In the 3.d audit, SAIs can ask how governments are acting to enhance capacity in some or all of the following, depending on their own national context and risk:

- forecasting, preventing and preparing for public health emergencies (PHEs) and threats
- adapting, absorbing and responding to PHEs and threats
- maintaining essential health services in all contexts (including during emergencies/crises).

The audits are expected to highlight current capacities of health systems resilience; the extent to which a whole-of-government approach and policy coherence have been utilised; and government efforts related to multistakeholder engagement and leaving no one behind in building health systems resilience related to progressing towards achieving the national target linked to 3.d by 2030. An overall positive achievement noted was that undertaking a complex health audit in the middle of a pandemic is possible and can contribute to building health systems resilience and recovery efforts. In their review of audit plans, draft summaries, and other work by the SAIs, both WHO and IDI have observed that SAIs have used the training and supplementary materials and applied various parts of it in their audits. This collaboration also demonstrates key considerations needed for successful partnership across multisectoral partners at global, regional and national levels. Such considerations can be applied in different contexts, including socioeconomic and health system recovery, to ensure whole-of-society and whole-of-government action in building health systems resilience and monitoring and evaluation to maintain and accelerate progress towards the national target linked to SDG3.d, health security and universal health coverage (UHC), as well as broader socioeconomic development.

## Introduction

The INTOSAI Development Initiative (IDI) and the Health Systems Resilience team at the World Health Organization (WHO) have been collaborating in facilitating 40 Supreme Audit Institutions’ (SAIs)^1^ performance audits of strong and resilient national public health systems linked to the national target of SDG 3.d across Africa, Americas, Asia and Oceania. INTOSAI Development Initiative is an INTOSAI^2^ organ that supports capacity development of SAIs mainly in developing countries. In this context, IDI has provided support to SAIs in conducting the 3.d audits and engaged with WHO to provide technical support to SAIs.

The audits were conducted during the COVID-19 pandemic and in the context of health systems recovery with relevant lessons within and beyond the health sector. Each country has a Supreme Audit Institution whose job is to audit public funds as an effective, accountable and inclusive institution. SAIs are oversight bodies in their respective countries and effective external government audit by SAIs is a key component of public financial management (PFM) and good governance. SAIs can contribute to the quality of government engagement and better state-society relations through their work. SAIs can also be key stakeholders in implementing the SDGs by undertaking audits related to the government implementation of efforts to reach SDG targets.

Health system resilience is defined as the capacity of health actors, institutions, and populations to prevent, prepare for, absorb, adapt, respond, and recover when faced with a wide range of risks and shocks in a timely, effective, and efficient manner while maintaining essential functions and services in all contexts and informed by lessons from the experience, transform and improve, as necessary (1–3). Past and ongoing public health challenges have highlighted that lack of health system resilience has profound impact on population health (e.g., COVID-19 related and excessive deaths, disruption of essential health services), socioeconomic development (e.g., global recession, widespread loss of livelihoods and income, regressing gains made towards universal health coverage (UHC) and in other SDGs) (4). Building back better, more resilient health systems has been a global priority in the context of recovery from COVID-19, humanitarian crisis and other public health events (5–8). Resilient health systems have the necessary capacities for managing complex and diverse health challenges every country is facing.

The SDG 3.d audits are performance audits and follow the International Standards of Supreme Audit Institutions (ISSAIs) for performance audits. The content of the support rendered to SAIs within the multisectoral collaboration between IDI and WHO followed these standards and IDI’s SDG Audit Model (ISAM) (9). As per ISAM, a performance audit of SDG implementation is “an audit of the implementation of the set of policies that contribute to the achievement of a nationally agreed target linked with one or more SDG targets. It concludes on the progress made towards the achievement of the nationally agreed target; how likely the target is to be achieved based on current trends; and the adequacy of the national target in comparison with the corresponding SDG target(s).” Moreover, an audit of SDGs implementation needs to be conducted using a whole-of-government approach. It needs to conclude on the extent of coherence and integration in the implementation of policies and to the extent possible, the audit could include objectives and questions that allow the SAI auditor to conclude on leaving no one behind and multi-stakeholder engagement.

**Figure 1:**
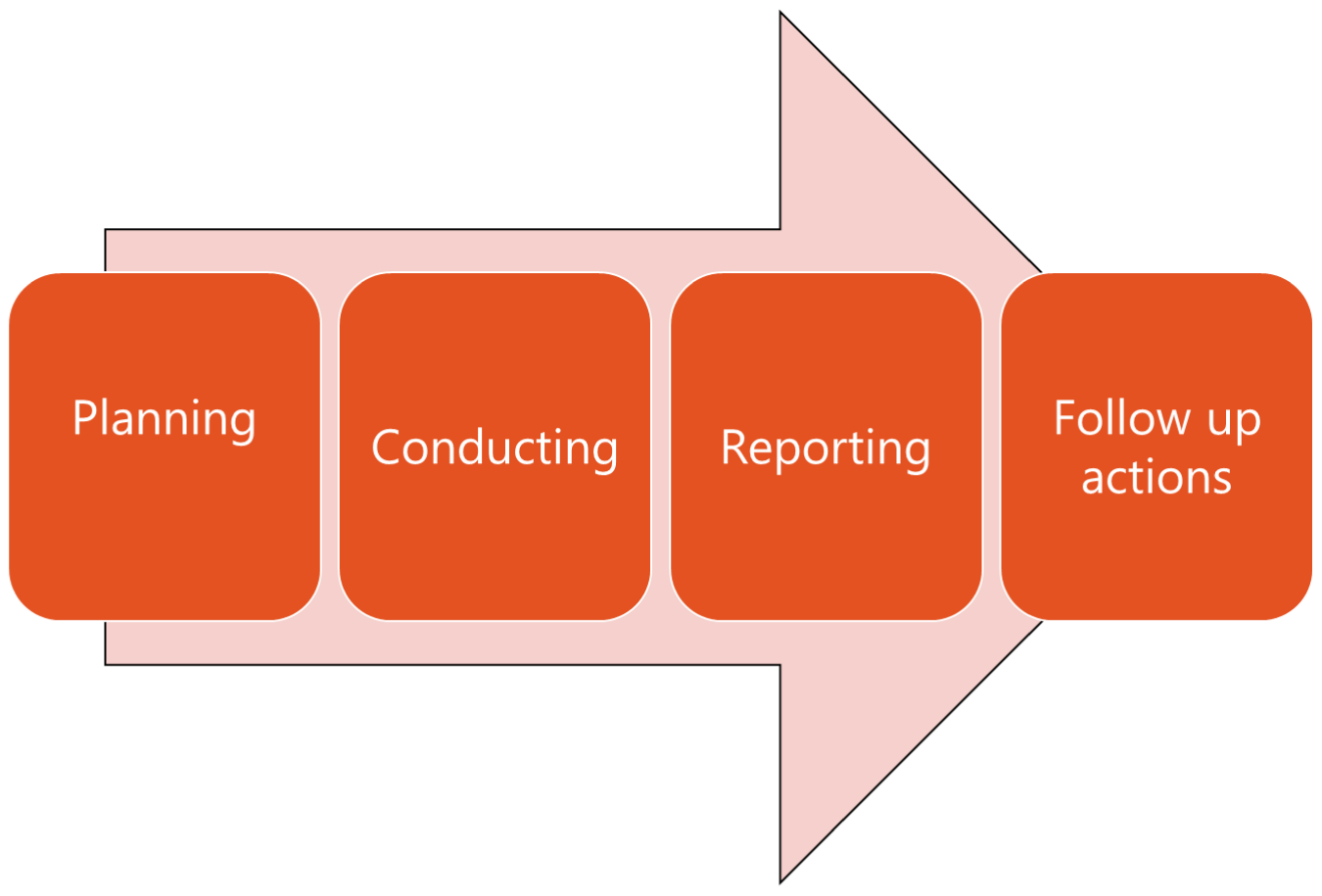
Process of performance audit defined by international standards.

While regular performance audits assess entities, projects, programmes or processes, the SDG audits, however, focus on the interplay between these components for achievement of cross-cutting results. Any performance audit that follows the international standards, include the following processes (Figure 1): (1) planning phrase often involving selection of topics and design of the audit; (2) conducting phase involving obtaining adequate and appropriate evidence to develop findings to answer the audit objectives and questions, conclusions and recommendations; (3) reporting phase involving preparing and developing an audit report to communicate audit results to the target audience; (4) follow-up actions on audit findings and recommendations to determine processes to address recommendations, assess if problems are resolved, and identify topics for future audits (10).

The collaboration between IDI and WHO covers the first three phases, as the follow up actions will happen after the reports have been published and will continue for two or 3 years pending on the nature of the recommendations in audit reports.

The objective of the collaboration between IDI and WHO was to facilitate the provision of technical expertise for integrated education on strong and resilient national public health systems in reference to the SAIs’ 3.d audits. By providing the technical support to IDI, WHO built the necessary capacity in IDI to support SAIs in exercising their follow up and review linked to SDG target 3.d. The aim of this article is to reflect on the project findings from the collaboration between IDI and WHO with a view to informing enhanced multisectoral action and policy options towards building health systems resilience and enhanced recovery, including from the perspective of the role of the supreme audit institutions in multisectoral collaboration efforts for building health systems resilience. The importance of multisectoral collaboration, communication and partnership is widely recognized for building health system resilience. However, our literature review found no focus on studying the role of audit institutions as a contributor to building health systems resilience through performance audits. Hence, this article represents a novel contribution in shedding light on SAIs’ important role in this area.

## Literature review

### The importance of multisectoral collaboration in building health systems resilience

A multisectoral approach to health can be understood as deliberate collaboration among various stakeholders both within and beyond the health sector, towards a shared vision on desired health and socioeconomic outcomes (11, 12). The importance of multisectoral collaboration, communication and partnership is widely recognized for building health system resilience (13–17). For example, Nabyonga-Orem et al. (18) found that stakeholder empowerment, competency development and proper information sharing are needed to strengthen policy dialogues between multisectoral actors across all levels in the context of Ebola outbreak. Moussallem et al. (19) found that the power relations between the health actors and stakeholders in other sectors affected the uptake of evidence in policy-making regarding Lebanese health system for the COVID-19 pandemic. However, there are not many studies examining the roles and mechanisms of specific non-health sectors in building health systems resilience. Response partners related to public health emergencies, communities, humanitarian support, and non-governmental organizations are the often-mentioned actors other than the health sector in the literature. Barker et al. examined how community engagement facilitates health systems resilience in low-resource settings during Ebola (20). Marome et al. (21) suggested the governments to strengthen governance across national to community levels for resilience engaging with multisectoral stakeholders including grassroots and community networks. The limited findings on incorporating actors out with the health sector is not altogether surprising as the concept of health system resilience is relatively new and not widely understood beyond the health policy and systems community. Furthermore, how to operationalise health systems resilience with multisectoral collaboration consideration is not well described in the literature (22–24). Ling et al. (25) suggested to gather evidence from organisations and individuals in other sectors that are more separated from health activities to assess how to maintain essential functions and services for health systems resilience. Resilient health systems can meet population health needs in both “peace” and emergency contexts; nevertheless, most of these studies have been conducted in the context of the Ebola epidemics, refugee crises and the COVID-19 pandemic.

### Lack of studies of auditing communities’ role in building health systems resilience

Monitoring and evaluation and accountability mechanisms are key to build health systems resilience. Woodward et al. (26) identified actors and accountability as a key research agenda in health system resilience. A Supreme Audit Institution (SAI) is a public body of a state or a supranational organization, exercises, by virtue of law, or other formal action of the state/the supranational organization, the highest public auditing function of that state/supranational organization in an independent manner (27). SAIs been an important factor in country’s accountability systems within and beyond the health sector. Their roles are traditionally known for the oversight of public financing; but SAIs are increasingly taking on performance auditing, which is defined as “an independent, objective and reliable examination of whether government undertakings, systems, operations, programmes, activities or organisations (28). Such audits to assess the government’s efforts at implementation of SDG commitments demonstrate SAIs’ expanding role and functions in the attainment of SDGs. SAIs’ audits of health systems resilience and important health-related SDGs such as UHC can be a powerful process to monitor and promote governments’ actions for achieving SDGs by 2030. However, in our searches of peer reviewed literature databases, we did not come across any literature with a focus on studying the role of audit institutions as a contributor to building health systems resilience through performance audits.

## Methods

With the aim to examine the IDI-WHO collaboration in health systems resilience and find out the role of the supreme audit institutions in multisectoral collaboration efforts for building health systems resilience, this paper draws on information and evidence from the collaboration between IDI and WHO in the SDG 3.d audit project. This project represents a multisectoral collaboration between the audit community and the international organizations of IDI and WHO in an SDG implementation audit.

For this paper, we draw on three sources of information throughout the SDG implementation audit process. First, the core IDI-WHO project team held live trainings and webinars and established an on-request communication channel, where the country-based audit team provided reflections and questions related to their understandings and application of health systems resilience in designing audit plans and reporting audit findings through focused-group discussions, surveys and question and answer sessions in an iterative base. Second, the core IDI-WHO project team provided ongoing written feedback on auditing team’s draft audit plans including audit design matrix, ecosystem mapping, risk profiling, as well as draft audit reports. IDI and its mentors also had frequent meetings with the audit teams to advise them in all processes of the audits. These audit plans and audit reports are not publicly available at this stage and therefore were not included in this article. Lastly, the core IDI-WHO project team provided both retrospective and prospective reflections on the innovative SDG audit model through semi-open discussions within the project team guided by guiding questions and in the project reporting.

These three sources of information can support the understanding of the process and impact of the multisectoral collaboration of IDI-WHO for building health systems resilience in recovery context. The information also enables to understand the audit sector’s role in multisectoral collaboration for building health systems resilience in many aspects, including improvement of conceptual understanding and prioritization of health systems resilience; identification of the baseline health systems capacities, strengths, gaps and needs; leveraging of strengths and opportunities and mobilization of support for building health systems resilience; creation of an enabling environment for health system resilience; and monitoring and evaluation of progress for evidence-informed follow-up actions. The authors analysed the qualitative data and identified emerging themes and key findings.

## Results: Modality of collaboration between IDI and who in audits of SDG 3.D linked to national public health system resilience

### Result 1: Collaboration provided a consolidated multisectoral overview and built audit teams’ knowledge base on an integrated approach to health systems resilience, needed for SAIs to audit their government’s efforts related to national public health systems resilience

The collaboration in audits of SDG 3.d started with professional education to build the knowledge of country-based Supreme Audit Institutions in the subject matter. IDI and WHO leveraged respective expertise in the design, development and delivery of 3.d Education content on Health Systems Resilience and SDG 3.d. WHO, as the subject matter expert, first developed a compendium of health systems resilience technical reference materials for self-learning, and IDI distributed technical materials to SAIs through IDI’s platforms and networks.

A fit-for-purpose training package on an integrated approach to building health systems resilience (linked to SDG 3.d) was subsequently developed based on online training on health systems resilience^3^ aimed to decision makers of health policies and managers of health services (29), and delivered in the format of four online interactive webinars and offline quizzes. The training material integrated key requirements, considerations, and general principles of building health systems resilience, e.g., multisectoral approach, public health-oriented planning, maintaining essential functions and services. IDI organized and mobilised a primary audience of over 130 auditors from SAIs in 40 countries as well as mentors from the regions from the participating SAIs.

The collaborative delivery of 3.d audit education contents on health system resilience leveraged the technical expertise of IDI and WHO, respectively. The compendium of resources aimed to support SAIs to familiarise with basic concepts and principals related to capacities of resilient health systems and the importance of health systems resilience in response to public health challenges. The training aimed to develop an in-depth understanding on the relationship between health systems resilience and SDG 3.d, requirements of building health systems resilience, key stakeholders, and assessment of health systems resilience, which are necessary for developing audit plans. Both education components supported SAIs at the audit planning stage as the critical considerations of building health system resilience are widely reflected in audit questions.

Collaborative activities on enhancing the knowledge base of SAIs continued after training sessions. Translating the general concepts of national public health systems resilience to what it implies in practice appeared to be challenging throughout the audit process but managed by collaborative efforts. For example, resilient health systems’ capacity to transform and improve informed by lessons from experiences was translated into audit questions and criteria such as: existence of national action plans to address the gaps identified in the International Health Regulations monitoring and evaluation and other health system assessment efforts, evidence of simulation exercises being conducted regularly, and evidence of after-action review or intra-action review being conducted. WHO continued to provide technical support through IDI in the format of document review, webinar and Q&A.

An overall positive achievement noted was that undertaking a complex health audit in the middle of a pandemic is possible and can inform health systems strengthening and recovery. In their review of audit plans, draft summaries, and other work by the SAIs, both WHO and IDI have observed that SAIs have used the training and supplementary materials and applied various parts of it in their audits.

### Result 2: The development and provision of the audit matrix by IDI and WHO facilitated a multisectoral approach to audit health systems resilience

Based on the audit design matrix reference provided by IDI, WHO and IDI co-developed a template of an audit design matrix for the 3.d audit. Audit objectives and questions are the foundation for an effective planning of any performance audit. Formulating objectives and questions requires to be based on key considerations for assessing progress of implementation of the nationally agreed target selected for the audit.

“A resilient health system is one that can prepare for, respond and adapt to disruptive public health events while ensuring the continuity of quality, essential health services at all levels of the health system” (3, 14). To support SAIs in formulating their audit questions suitable for national contexts, WHO developed a set of general questions in line with the capacities and attributes of resilient health systems for SAIs’ consideration and adaptation based on their national contexts and institute capacity (Box 1). Sub-questions which are more specific and manageable to answer were developed with a focus on the government’s compliance to SDG principles (e.g., leaving no one behind, whole-of-society engagement, policy coherence), general principles for building health systems resilience (e.g., public health orientated health system strengthening, all-hazard approach, applying an integrated approach to avoid, and perpetuate, fragmentation in health systems), and resilience building efforts at different policy stages (e.g., policy and planning, operationalisation and implementation, and assessment) (Box 1).

BOX 1. **Initial audit questions and sub-questions for SAIs’ consideration and contextualisation**

Audit objective 1: To what extent does the government strengthen health system’s capacities to forecast, prevent and prepare for public health risks building on emerging lessons learnt from recent public health events?

1.1 How is the government putting in place processes and institutional arrangements to take forward the lessons to enhance capacities to forecast, prevent and prepare for public health risks through the country’s legislation, policy, plans, budget and programmes, including the country’s existing sustainable development strategy, if there is one? Is the government putting in place covid policy framework, processes, and institutional arrangements (whole-of-government approach)?

1.2 How is the government ensuring inclusive, collective and whole-of-society approaches (all stakeholders) in building health system’s capacities to forecast, prevent and prepare for public health risks at all levels?

1.3 How does the government routinely assess its capacities to forecast, prevent and prepare for public health risks, in line with meeting SDG 3.d targets?

Audit objective 2: To what extent does the government take proactive measures drawn from lessons learnt from recent public health events, to strengthen health system’s capacities to adapt, absorb and respond to PHEs, while maintaining essential health services?

2.1 How does the government ensure a multi-sectoral coordination mechanism in place to adapt, absorb and respond to PHEs?

2.2 How does the government maintain essential health services, including adequately addressing the health needs of marginalised groups?

2.3 How does the government apply lessons from monitoring and evaluation processes to strengthen health system’s capacities to adapt, absorb and respond to PHEs?

Audit objective 3: To what extent does the government learn from recent public health events, to plan for health system recovery and transformation towards resilience?

3.1 How does the government learn from recent public health events and apply lessons learnt in reviewing, updating and aligning health system strengthening and health security institutional arrangements, strategies, policies, plans, and interventions?

3.2 How does the government ensure “sustainable development” and “building back better” principles applied in health systems recovery and transformation?

3.3 How does the government ensure adequate resources allocated for sustainable health system recovery and transformation towards resilience?

An audit design matrix is a tool for systemising the entire auditing process. The matrix often includes audit questions, criteria (i.e., the ideal situation in relation to the audit questions), and methods (i.e., how the audit team assesses the audit questions in relation to criteria) as main elements connected as a logical chain of reasoning (30, 31). The matrix must be developed for all sub questions. IDI and WHO took the approach of co-developing the matrix by leveraging each other’s comparative organisational advantages and technical expertise in the subject matter and audit, respectively. The matrix provided a generic example that was customised by SAIs to fit their national context and scope of the audit.

Audit criteria were developed based on requirements of building health systems resilience linked to SDG 3.d, in aspects of institutional arrangements for resilience building, accountability of health authorities and allied sectors, integration and coherence of health sector policies including those with a focus on health security, dedicated consideration of vulnerable and marginalised populations and communities in health system strengthening, comprehensive mechanisms for identifying and utilising lessons from public health events to improve health systems and sustainable resources for health system resilience building.

Building back better and more resilient health systems is not solely the responsibility of the health authority but requires coordinated whole-of-society efforts. Therefore, in the methods, recommended sources of information span from traditional health actors (e.g., ministries of health and national public health institutes) to actors in other sectors who contribute to health system recovery and resilience (e.g., ministry of finance, disaster management agencies, the private sector). Information from a wide range of sources would allow triangulation and verification of whole-of-governments’ and -societies’ actions and commitments.

### Result 3: Stakeholder mapping is useful for identifying national and local level players involved in building public health systems resilience, supplementing the ecosystem and enables an assessment of the government’s multistakeholder engagement

As an SDG audit, by definition, should include objectives and questions that allow the SAI auditor to conclude on multi-stakeholder engagement by the government, IDI provided the teams with examples of stakeholder analysis and RACI analysis (i.e., responsible, accountable, consulted, and informed) in the ISAM guide (9). These supplemented the eco-system map that WHO provided (described in “Result 4”) and the tools have been widely used by the audit teams. Stakeholder mapping can form an integral part of an ecosystem map as a first step and can also be used as input to initiate the assessment of whole-of-government and -society efforts to engage stakeholders in implementation of any SDG goal or target, as it provides an overview of who is involved and their interests in the area being audited.

### Result 4: The IDI-WHO collaboration fostered application of a whole-of-government and whole-of-society approach to auditing the SDGs through an ecosystem approach and map

**Figure 2:**
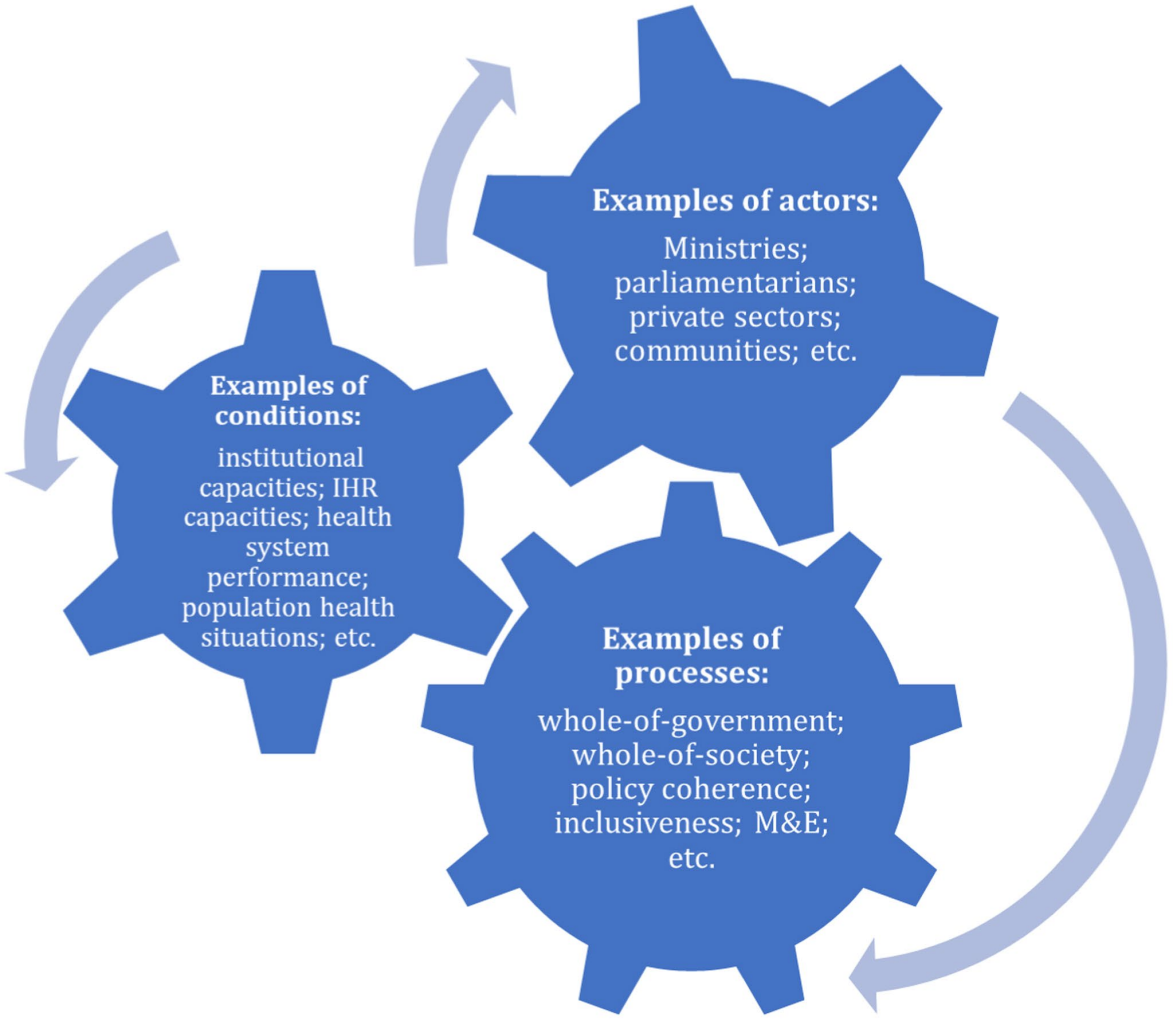
Ecosystem map in relation to health systems resilience with examples.

After designing the audit design matrix, conducting a stakeholder mapping and making an audit plan, SAIs start approaching key actors to gather information to answer audit questions. Operationalising health systems resilience requires using a system and multisectoral approach with public health underpinning (3, 32). To support SAIs to better understand the dynamics in health systems resilience and map and access the key actors and sources of information for this audit, an ecosystem map is utilised as a tool for SAIs to understand interconnected and interdependent actors for health agendas (e.g., ministry of health, other ministries like agriculture and transportation, national and local parliamentarians, international agencies, communities); conditions underlying context in the health system and wider society (e.g., broader determinants of health, available recourses and baseline health system capacities, current risk and vulnerable profiling); and processes that indicating how actors interact in policy and planning, implementation and monitoring and evaluation (e.g., intersectoral fora, national priority setting, after action reviews), that are crucial in building health system resilience to meet SDG 3.d and other SDG targets. Multisectoral collaboration and conducive legislative and policy environments are examples to enable and maintain a healthy ecosystem for health system resilience and recovery. WHO supported SAIs by developing an example of an ecosystem map for SAIs to consult in making their own map. The map was based on the three elements of conditions, actors and processes (Figure 2).

### Result 5: Collaboration for agile technical support provided from WHO to IDI and SAIs enabled SAIs to audit a new technical area and helped timely identification and clarification of misunderstanding on applying health system resilience concepts in audits of SDG 3.d

As SAIs have planned and conducted the 3.d audits, WHO provided follow-up targeted technical support and expertise on health systems resilience, including both on-request support in the process of national audits, through dedicated webinars, reviews (including review of audit design matrix, audit plans and draft audit reports), and *ad-hoc* responses to SAIs’ technical questions on resilience; and on-demand technical support materials based on the needs of SAIs that are commonly or frequently raised in the process of national audits. This is complementary to planned training sessions to all SAIs and in response to country-based SAIs’ specific needs as each audit teams adopt different audit objectives and plans.

The FAQ document and webinar session and agile support to audit teams help address and clarify key conceptual and operational aspects of health systems resilience. For example, one misconception was that “strong health systems” are always resilient. However, many health systems that have been seen as strong do not necessarily possess the attributes to be resilient to disruptive public health events (such as those of many high-income country health systems during COVID-19) and chronic stressors (such as health systems’ capacities to meet the needs of growing and evolving population demographics) (2, 33). Another misconception was that building resilience requires excessive costs. However, health system resilience is not attributed to resource levels but how well the available resources are used to intentionally design, orient, and develop the health system (5). There was also a wider misapprehension that resilience is only relevant to emergency or acute situations; however, a resilient health system is that which can perform its functions both within and beyond the contexts of shock events. Resilient health systems are capable of responding to both acute and chronic shocks as well as everyday challenges to the health system (e.g., payment delays, unpredictable staff and evolving patient and community expectations). Through tackling these misconceptions in audit institutions through agile and timely technical support, SAIs were equipped to conduct SDG 3.d audits as well as playing a stronger role in advocating key messages to national governments and other multisectoral actors. Furthermore, as a result, SAIs are also better equipped to play a key role in audit follow-up and thereby contributing to long term, sustainable building of health systems resilience with broader societal benefit (e.g., reduced excess mortality and morbidity and socioeconomic disruption).

Altogether, the support provided through the multisectoral collaboration equipped SAIs to undertake an audit of a new technical area demonstrating the mutual benefit and added value to society of multisectoral and multi-actor collaboration.

### Result 6: Audits provide key evidence to inform resilience building

SAIs through conducting the 3.d audits can also provide critical evidence to inform what works and what needs to be improved in building health systems resilience. While the concept of resilience is widely appreciated and supported with a rapidly growing knowledge base and in global and national health declarations, resolutions, and strategies, it requires further clarity for countries in terms of how to operationalise resilience at national and subnational levels and for global actors to support countries. Since COVID-19, there have been heightened political and public attention as well as the need for operational clarity in support of socioeconomic and health system recovery. SAIs conducting the 3.d audit provide valuable first-hand information on what countries are doing to build health systems resilience, what is going well and what major gaps exist. For example, the preliminary audit reports show that audits conducted usually identified duplication, fragmentation, gaps and overlap across health and allied sectors’ policies, planning and programmes for population health. The case examples and identified gaps can inform national governments’ policymaking as well as global actor’s strategic direction setting. This is especially the case in the context of participating SAIs which cover countries facing frequent and severe public health challenges relating to conflicts and climate change or natural disasters. Their audit reports contribute to the evidence base informing national and global actors’ targeted support to build health system resilience in these vulnerable countries, such as small-island developing states, and countries in fragile, conflict-affected, and vulnerable settings.

### Result 7: SAIs can contribute to resilient recovery in their forward-looking orientation of the audits—supporting government efforts in building back better and build health systems resilience going forward

Performance audits are usually backward-looking in nature, as they assess government performance in implementing efforts in an area/project/programme/entity. With their focus on current efforts by governments to strengthen health systems resilience based on lessons learnt from previous pandemics, the 3.d audits are forward-looking. ISSAI 300 calls for dialogue with audited entities and relevant stakeholders from the start of the audit. As to the reporting phase of a performance audit, “audited entities should be given an opportunity to comment on the audit findings, conclusions and recommendations before the SAI issues its audit report” (28). In the SDG 3.d audits, this dialogue with ministries of health and other involved ministries/agencies/other public sector bodies involved in efforts related to national public health systems resilience can inform ongoing policymaking and implementation in this area. The findings and recommendations of these audits may thus contribute to strengthening the resilience of public health systems to current and future threats, providing that the audited entities act on these findings and recommendations.

## Discussion: Future impact of 3.d audit and SAI contribution to health systems resilience in recovery context

As audit impact is a shared responsibility among SAIs and its ecosystem, SAIs rely on the uptake of the audit report findings and recommendations by other stakeholders for the reports to contribute to impact. As the 3.d audits are forward-looking in nature, there is an immense potential for governments, development partners and other stakeholders engaged in health systems strengthening efforts at the country, regional and international levels to use the reports in taking stock of the current situation and consider how they may best follow up on the recommendations of the audit reports, given their role and responsibilities in protecting and promoting population health and wellbeing, at their respective levels.

As per the performance audit standards, the “auditor shall provide constructive recommendations that are likely to contribute significantly to addressing the weaknesses or problems identified by the audit, whenever relevant and allowed by the SAI’s mandate” (34). Recommendations will differ among the 3.d audits as the situation found in the countries varies. However, they will all deal with relevant aspects to be addressed to create more resilient health systems, with a focus on whole-of-government efforts. Hence, the recommendations may potentially influence cross-country and region learning and future policies, e.g., in terms of these becoming more multisectoral in character. As some SAIs undertake follow up actions on the audit reports after a certain period, the reports may influence future policies as governments know that they will be held to account for their actions in implementing efforts to meet the audit recommendations.

Moreover, audits in the health sector may have a deterrent effect on negative government actions within the sector, as governments may anticipate that SAIs will audit areas with specific high risks and issues, thus making governments act to address and mitigate such risks to avoid an audit in the first instance.

As the recipients of the 3.d audit reports in many of the countries involved in this audit, parliaments also have a role to play in the accountability chain. Parliaments may request audited entities to act upon the recommendations and follow up on their implementation of the recommendations in later parliamentary debates and through other follow-up measures. Multisectoral fora at national and subnational levels can also be leveraged to promote and sustain the audit impact to build health system resilience in tackling shock events as well as during periods of relative normalcy to enable better resilience to future public health events.

Supreme Audit Institutions have a role to play in *other* health areas as well through conducting performance audits of other SDG 3 targets or by undertaking regular performance audits on health (outside the SDGs). A related target that would be relevant for future audits is UHC given that such coverage constitutes part of a resilient health system and is a global health priority. Financial audits and compliance audits are also relevant audits that may contribute to a more resilient health system. In general, by exercising their oversight functions through auditing the area of health, whether it is performance, financial or compliance audits, SAIs may contribute to more efficient resource allocation and use in the health sector, improved performance of health sector interventions and adherence to laws and regulations relating to health. While there are a plethora of topics and approaches that SAIs may audit and the relevant themes will depend on the country context, all SAIs have a significant role to play in their respective countries—shedding light on existing deficiencies that hamper health sector resilience.

Participating SAIs are investing their resources in learning from global knowledge of health systems resilience and applying the knowledge in their audits. SAIs have formulated value-added recommendations, such as those relevant to investing in strengthening foundational health system capacities for resilience, defining clear roles and responsibilities in government structures for health system resilience, and mobilising and utilising whole-of-government and whole-of-society efforts and resources for health system resilience. These audit recommendations facilitate translating and bringing global knowledge to national contexts, which can inform governments’ high-level decision-making to make sustainable impact. For example, it is important for national actors to understand that resilience is not merely a biproduct or an inevitable outcome of any investment in the health sector; resilience must be proactively and intentionally programmed into health systems strengthening and other complementary efforts such as those targeting health security, specific diseases, life-course-related and environmental issues.

The SDG 3.d audit linked to health systems resilience can be seen as one novel approach contributing to the monitoring and evaluation of the government’s commitment and actions to building health systems resilience. SAIs’ current and potential future audits of health systems resilience and important health-related SDGs such as UHC can be a powerful process to monitor and promote governments’ actions for achieving SDGs by 2030. There is a gap in measurement and monitoring mechanisms of health systems resilience (35, 36); SAIs conducting this audit could support the trending global acknowledgement of the importance of measuring and monitoring health systems resilience, where different global actors are forming technical collaboration to reach global consensus, inform country-focused support, and advocate for government’s actions.

The SAIs brings audit as an accountability mechanism to whole-of-government efforts in strengthening health systems. Multisectoral accountability mechanisms are often lacking in promoting and protecting population health (37). Such an accountability mechanism is key to ensure sustained whole-of-government approach to health/health-in-all-policies approach for resilient health systems. In the recovery context, and with resource restraints, all ministries must work together, often pooling resources, and put health at the centre in recovery efforts due to health’s fundamental role in the normal functioning of society, travel and trade. This is complementary to country’s self-assessment and reporting. With a pandemic treaty on the way, WHO’s position paper on building health systems resilience, and other global and state commitments to population health, accountability is key to translating commitments into sustained and integrated and coherent actions.

Building health system resilience is pertinent in the recovery context. Health should not be viewed as a cost but as an investment by policymakers. An additional investment of one dollar per person per year in the prevention and treatment of noncommunicable diseases, could save 7 million lives in low- and lower-middle-income countries (38). Health is at the centre of recovery efforts. Lessons identified in COVID-19 highlight there is no socioeconomic development if population health cannot be protected. However, health is often underinvested in and where investments and efforts are made, they can be fragmented (39–41). Audits of health-related SDG targets could strengthen the accountability of the government to public health. As the audits have applied a whole-of-government approach to examine health systems resilience, they could also contribute to government efforts at enhancing multisectoral coordination for a less fragmented future approach to preparedness for future public health risks.

Despite health system resilience being increasingly discussed as a concept since the Ebola outbreaks in 2014–2016 and more recently during the COIVD-19 pandemic, operationalising health system resilience remains a challenge. SAIs required support in understanding the concept of health system resilience in the context of implementation and monitoring. Some common misconception includes that building health system resilience requires high costs; health system resilience is only relevant to emergency preparedness and response; and a strong health system is always resilient. WHO reemphasized key messages, such as health system resilience is not attributed to resource levels but how well the available resources are used to intentionally design, orient and develop the health system; resilience must enable response to both acute and chronic shocks and everyday challenges to the health system; and many health systems that have been perceived as strong do not necessarily develop attributes to be resilient to disruptive public health events and chronic stressors. The process of tackling these challenges through education (e.g., training sessions), two-way communication (e.g., interactive webinars and Q&A sessions), and engagement (e.g., exercises and practices in developing and applying audit design matrix) could shed light towards the operationalisation of health system resilience within and beyond the health sector.

Moreover, as SAIs can follow up on the implementation of audit recommendations, the reports and follow up of them provide an instrument for taking stock of any improvements in a government’s efforts related to health systems resilience to meet the SDG 3.d target by 2030.

As SAIs’ audit reports are an independent and authoritative source of information, WHO and other international organisations at the national and international level may also take a multisectoral view by utilizing these audit reports on health systems resilience as well as other audit reports on health in the health sector assessments they regularly undertake.

Audits can furthermore serve as an accountability or monitoring and evaluation mechanism supporting the global health agenda in recovery from COVID-19 and other crises as they are external reviews by an independent public oversight body at the country level. Furthermore, the multisectoral collaboration as seen between IDI and WHO providing support to the SAIs in undertaking such audits facilitates knowledge transfer from international to national levels. WHO Director-General’s outlines five priorities for the world and for WHO going forward at the 150th session of the Executive Board (42), including making an urgent paradigm shift towards promoting health and well-being and preventing disease by addressing its root causes, a radical reorientation of health systems towards primary health care, as the foundation of UHC, and harness the power of science, research innovation, data and digital technologies as critical enablers. This necessitates monitoring and evaluating governments’ commitments and actions in these health areas. Resilience is built over time and requires intentional design to health systems and multisectoral efforts. Auditing health-related SDG targets and facilitating audit impact of such audits by other players at national and international levels will strongly support government planning, financing and implementing health and intersectoral strategies for health.

## Conclusion

Multisectoral collaboration is essential to meet global health goals in times of normalcy as well as during periods of emergency or crisis. The need for multisectoral collaboration at the country level and at the international level is critical to building and sustaining health systems resilience for UHC, health security and the SDGs. Lessons from health systems shocks highlight the need to position health as central to national agendas for socioeconomic development, with participation of all sectors because when health is affected, everything is affected. As key independent external oversight bodies of government funds, SAIs can play a critical role in recovery and in building future health systems resilience across sectors. SAIs can contribute by issuing independent audit reports that assess the performance of government efforts and by following up on the implementation of the recommendations in such reports after some time. However, potential impact generated from the audits is a shared responsibility among SAIs, audited entities, legislative bodies, civil society organisations and other stakeholders in the country context in which the SAI operates. WHO and other international, regional, national and local stakeholders may use the audit reports as a valuable authoritative source of independent information about the status of national public health systems resilience in the respective countries. They may also try to facilitate audit impact of the reports by acting on the recommendations wherever applicable to them. As the world moves towards recovery after the pandemic, multisectoral collaboration across all levels remains pertinent for creating a path towards resilient national public health systems and making and sustaining progress towards the SDGs and key global health goals.

## Data availability statement

Requests to access data presented in this article should be directed to saikats@who.int.

## Author contributions

SH, SM and YZ contributed to the conception, design of the research and developed the first draft of the manuscript. SH, SM, YZ, SS and AS collected information and conducted the analysis. SS reviewed the manuscript and provided key inputs on health systems resilience. SH and SM coordinated inputs from all authors. All authors contributed to the article and approved the submitted version.

## Conflict of interest

The authors declare that the research was conducted in the absence of any commercial or financial relationships that could be construed as a potential conflict of interest.

## Publisher’s note

All claims expressed in this article are solely those of the authors and do not necessarily represent those of their affiliated organizations, or those of the publisher, the editors and the reviewers. Any product that may be evaluated in this article, or claim that may be made by its manufacturer, is not guaranteed or endorsed by the publisher.

## Author disclaimer

The perspectives expressed in this article are those of the authors and do not necessarily represent the decisions or the policies of the World Health Organization or of IDI.

## Footnotes

^1^Participating SAIs include Algeria, Aruba, Bahamas, Bangladesh, Bhutan, Egypt, Fiji, Grenada, Guyana, Indonesia, Iraq, Jamaica, Lao PDR, Libya, Malaysia, Mongolia, Montserrat, Morocco, Myanmar, Nepal, Oman, Pakistan, Peru, Philippines, Palestine, Saint Lucia, Saint Vincent and The Grenadines, Sri Lanka, Syria, Tajikistan, Thailand, Tonga, Trinidad and Tobago, Tunisia, and Sudan.

^2^The INTOSAI stands for the International Organization of Supreme Audit Institutions and is a membership organisation of 194 SAIs from all over the world. INTOSAI recognised the importance of the 2030 Agenda for Sustainable Development and included SDGs as cross cutting priority in its Strategic Plan 2017–2022. IDI collaborated with the INTOSAI Knowledge Sharing Committee (KSC) and INTOSAI Regions (ASOSAI, ARABOSAI, CAROSAI, PASAI) and other key stakeholders to support a cooperative audit of strong and resilient national public health systems (linked to SDG target 3.d).

^3^Online training: An integrated approach to building health systems resilience. Available at: https://openwho.org/courses/health-service-resilience.
